# The effect of “universal test and treat” program on HIV treatment outcomes and patient survival among a cohort of adults taking antiretroviral treatment (ART) in low income settings of Gurage zone, South Ethiopia

**DOI:** 10.1186/s12981-020-00274-3

**Published:** 2020-05-18

**Authors:** Tadele Girum, Fedila Yasin, Abebaw Wasie, Teha Shumbej, Fitsum Bekele, Bereket Zeleke

**Affiliations:** 1 Department of Public Health, College of Medicine and Health Sciences, Wolkite University, Wolkite, Ethiopia; 2 Department of Medical Laboratory Science, College of Medicine and Health Sciences, Wolkite University, Wolkite, Ethiopia; 3 Department of Pharmacy, College of Medicine and Health Sciences, Wolkite University, Wolkite, Ethiopia

**Keywords:** Universal test and treat, Differed treatment, Patient survival, HIV treatment outcome

## Abstract

**Background:**

Through universal “test and treat approach” (UTT) it is believed that HIV new infection and AIDS related death will be reduced at community level and through time HIV can be eliminated. With this assumption the UTT program was implemented since 2016. However, the effect of this program in terms of individual patient survival and treatment outcome was not assessed in relation to the pre-existing defer treatment approach.

**Objective:**

To assess the effects of UTT program on HIV treatment outcomes and patient survival among a cohort of adult HIV infected patients taking antiretroviral treatment in Gurage zone health facilities.

**Methods:**

Institution based retrospective cohort study was conducted in facilities providing HIV care and treatment. Eight years (2012–2019) HIV/AIDS treatment records were included in the study. Five hundred HIV/AIDS treatment records were randomly selected and reviewed. Data were abstracted using standardized checklist by trained health professionals; then it was cleaned, edited and entered by Epi info version 7 and analyzed by STATA. Cox model was built to estimate survival differences across different study variables.

**Results:**

A total of 500 patients were followed for 1632.6 person-year (PY) of observation. The overall incidence density rate (IDR) of death in the cohort was 3 per-100-PY. It was significantly higher for differed treatment program, which is 3.8 per-100-PY compared to 2.4 per-100-PY in UTT program with a p value of 0.001. The relative risk of death among differed cases was 1.58 times higher than the UTT cases. The cumulative probability of survival at the end of 1st, 2nd, 3rd, and 4th years was 98%, 90.2%, 89.2% and 88% respectively with difference between groups. The log rank test and Kaplan–Meier survival curve indicated patients enrolled in the UTT program survived longer than patients enrolled in the differed treatment program (log rank X^2^ test = 4.1, p value = 0.04). Age, residence, base line CD4 count, program of enrolment, development of new OIS and treatment failure were predicted mortality from HIV infection.

**Conclusion:**

Mortality was significantly reduced after UTT. Therefore, intervention to further reduce deaths has to focus on early initiation of treatment and strengthening UTT programs.

## Background

Human immunodeficiency virus (HIV) infection remains the leading cause of morbidity and mortality throughout the world. Ethiopia is one of HIV hard hit countries with a prevalence of 1.1% [1, 2]. World Health Organization (WHO) developed the “universal test and treat” (UTT) program as strategy for HIV elimination in place of the previous “differed treatment” (CD4 based and WHO clinical staging approaches) program [3–6]. UTT is a program which commends all population at risk is screened for HIV infection and those diagnosed HIV positive receive early treatment regardless of their CD4 count and WHO clinical stage. Many countries including Ethiopia had adopted the ‘test and treat’ program [7–9].

Although, the health care systems has accepted the public health benefit of universal test and treat strategy for the prevention of new transmission, evidences on its impact on clinical outcome and patient survival are limited [3]. Although, WHO recommends the universal test and treat program, about 11% of low and middle income countries do not implemented it yet [10]. On the contrary, there are countries that have implemented the universal test and treat program before the WHO recommendation by their own initiative. As a result of clinical, public health and economic concerns different countries have been recommended ART initiation at different stages of the disease or at different levels of CD4 count [11, 12]. Thus, assuring the individual level benefit of the program in terms of treatment outcome and patient survival is very important to bring additional evidence helpful for scaling up program intervention.

Few studies conducted abroad in areas of treatment outcomes have reported different findings. Some model studies had shown that test and treat strategy as the effect of early initiation of treatment has impact on all epidemiological aspects of HIV/AIDS. The effects reported and predicted were particularly related with achieving higher survival time, development of resistance at higher rate, higher immune reconstitution syndrome rate and lower mortality. However, there is shortage of clinical research for clinical decisions [4–6, 8, 9].

A research in Canada has shown that test and treat strategy have associated with decreased morbidity, mortality and HIV transmission, and increases the life expectancy of people living with HIV/AIDS (PLWHA) [13, 14]. Another study on early initiation of treatment has shown to reduce transmission to the HIV negative partner by 96% and reduces adverse health events by 41% for the person living with HIV [15, 16]. Furthermore, a research from South Africa indicated that, implementation of universal testing and treatment initiation for adults over 15 years old would subsequently decrease HIV prevalence by reducing rate of transmission [17].

Universal testing and treatment alone was associated with significant gain in life which is estimated 12.0 (11.3–12.2) months, In addition it results in 27.7% decrease in deaths from HIV and 1.6% reductions in adult HIV prevalence compared to the differed treatment program [18]. Recent two randomized studies showed that ART initiation immediately after HIV diagnosis irrespective of the CD4+ T cell count leads to a significant reduction of morbidity and mortality [15, 17–21]. It can also improve the treatment outcome of HIV infected patients by increasing uptake of the therapy and reducing lost to follow-up [20–23].

On the other hand possibility of poor ART adherence due to rapid ART initiation and shorter counseling time, pill burden due to other concurrent comorbidities; and presence of immune reconstitution inflammatory syndrome (IRIS) in patients with low CD4 level, especially in individuals with advanced disease raised concerns in the program [23–26]. On top of that, researches have reported that asymptomatic patients with higher CD4 cell counts has poor adherence to ART so that early initiation would results in loss to follow up [25]. Moreover, widespread use of antiretroviral treatment at a population and individual level may lead to development of drug resistance [26, 27].

Despite all associated concerns, the UTT program has been in practice since 2016 in Ethiopia. It is partly on implementation to reduce transmission of the disease at community level. However, its real effect on the treatment outcome and patient survival was not evaluated. Therefore, this study is aimed to assess the effect of the UTT program in treatment outcome and patient survival, by recruiting a cohort of ART users in the new (UTT) approach and the previous (differed treatment/CD4 based) programs in Ethiopia, Gurage zone. The evidence will be used as base line information for planners, implementers and aid organizations.

## Methods and materials

### Study design and settings

This institution based retrospective cohort study was conducted in health facilities of Gurage zone, Southern Ethiopia from May/2019 to June/2019 by using 8 year cohorts. The zone has 13 districts and 5 town administrations. There are 74 health centers, 6 hospitals and 4 private clinics. Of these 20 facilities provide HIV care and treatment in the area. There are clients initially enrolled in the differed treatment and the current UTT programs.

### Study population and sampling technique

The source population was all adults (age 15+) with HIV enrolled to treatment program in all health facilities of Gurage zone. Sample size is calculated based on, sample size estimation for the assessment of survival time under the Cox proportional hazards model/log rank test by using the STATA Version 11.0 computer program considering the following assumptions: hazard ratio of 0.77 [20], 60% proportion of controls, 0.5 standard deviation of covariates of interest, with 5% marginal error and power of 80%. Finally by adding 10% for incompleteness, the sample size was 512. The sample was allocated proportionally for the five selected facilities and records were selected randomly.

### Data collection procedure and data quality control

The sources of data for this study were Pre-ART register, the ART register and the patients’ ART follow up and medical charts. In those registers and follow up charts, clients’ socio demographic, clinical and laboratory information, treatments being provided, the follow up status of each client were recorded. Data was collected from client charts using a structured checklist for records review developed from the registers and follow up charts. Eight data collectors and six supervisors who are health professionals and working in ART clinics were recruited for data collection after getting training on the tool.

### Study variables and data analysis

The outcome variable is time to death from enrolment to the cART program. The survival time is measured as the time period between date of enrolment and date of death, and it is dichotomized as death and censored. The censored cases include the alive patients, defaulters and transferred outs.

Data was cleaned, coded and entered into Epi-info version 7 and exported to STATA version 11, and then exploratory data analysis carried out to check assumptions. Kaplan–Meier survival curve together with log rank test was fitted to test for the presence of difference in survival time and incidence of death among patients enrolled in the UTT and differed treatment programs (UTT and differed). Incidence of death with respect to person time at risk was calculated. Finally, Cox-regression analysis was carried out to identify independent predictors of death in both groups. The forward stepwise regression method was applied and level of significance was used at p value less than 0.05. Model fitness checked by graphing residual plots with Cox-snell residual plot.

## Results

A total of 500 randomly selected ART records (204 from the test and treat program and 296 from deferred treatment/CD4 based treatment program) were extracted with structured check list. Two third (67.2%) of the patients enrolled into the study were females and 280 (56%) were urban residents. Nearly half (52.8%) of the clients were married and one third (36.8%) of patients has no formal education (Table 1).

**Table 1:** Socio-demographic and baseline clinical information of clients in the cART, Gurage zone, 2019

Variables	Outcome by program				
	UTT (N, %)		CD4 based (N, %)		Total
	Died	Censored	Died	Censored	(N, %)
Mean age at DX	35 ± 8.9		35.4 ± 9.3		35 ± 9.3
Mean weight	53 ± 12		51 ± 10		52.16 ± 11.2
Sex					
Male	4 (33.3)	76 (39.6)	16 (44.5)	68 (26)	164 (32.8)
Female	8 (66.7)	116 (60.4)	20 (55.5)	192 (74)	336 (67.2)
Residence					
Rural	8 (66.7)	58 (30.2)	28 (77.8)	126 (48.5)	220 (44)
Urban	4 (33.3)	134 (69.8)	8 (22.2)	134 (51.5)	280 (56)
Marital status					
Single	2 (16.7)	26 (13.5)	2 (5)	48 (18.5)	78 (15.6)
Married	4 (33.3)	110 (57.3)	18 (50)	132 (50.75)	264 (52.8)
Divorced	6 (50)	56 (29.2)	16 (45)	80 (30.75)	158 (31.6)
Educational status					
Illiterate	10 (83.3)	64 (33.3)	16 (44.5)	94 (36)	184 (36.8)
Literate	2 (16.7)	128 (66.7)	20 (55.5)	166 (64)	316 (63.2)
WHO stage					
Stage I	0 (0)	80 (41.6)	4 (11.1)	34 (13)	118 (23.6)
Stage II	4 (33.3)	64 (33.4)	0 (0)	60 (23)	128 (25.6)
Stage III	8 (66.7)	36 (18.75)	30 (83.3)	156 (60)	230 (46)
Stage IV	0 (0)	12 (6.25)	2 (5.6)	10 (4)	24 (4.8)
Median CD4 count	257.5 (IQR: 129–560)		182 (IQR: 110–234.5)		198 (IQR: 125–302)
Median time from diagnosis to Rx	0.3 (IQR = 0.1–4.1)		0.9 (IQR = 0.6–1.5)		0.7 (IQR = 0.2–1.1)
OIS					
Yes	4 (33.3)	74 (38.5)	24 (66.7)	140 (53.8)	242 (48.4)
No	8 (66.7)	118 (61.5)	12 (33.3)	120 (46.2)	258 (51.6)
New OIS					
Yes	6 (50)	18 (9.4)	6 (16.7)	10 (3.84)	40 (1.6)
No	6 (50)	174 (90.6)	30 (83.3)	250 (96.16)	460 (98.4)
Treatment failure					
Yes	8 (66.7)	10 (5.2)	10 (27.8)	2 (0.8)	30 (6)
No	4 (33.3)	182 (94.8)	26 (62.2)	258 (99.2)	470 (94)
Treatment switch					
Yes	0 (0)	2 (1)	2 (5.5)	4 (1.54)	8 (1.6)
No	12 (100)	190 (99)	34 (94.5)	256 (98.46)	492 (98.4)

The mean age at time of diagnosis was 35 (SD = 9.3) years with no difference between the two programs. The median time from diagnosis to initiation of treatment was 0.7 (IQR = 0.2–1.1) year. The average weight of participants was 52.16 kg (SD = 11.2), patients in the UTT program have slightly higher weight (53 ± 12 kg) than patients in the differed program (51 ± 10 kg). The median CD4 count during initiation of ART was 198 (IQR: 125–302), it was higher among patients in the UTT program 257.5 (IQR: 129–560) than the differed treatment 182 (IQR: 110–234.5) (Table 1).

During initiation of ART 50.8% of the patients were in WHO clinical stage III and IV in both groups. Specifically on the UTT program, only 27.5% of patients were in WHO clinical stage III and IV, whereas in the deferred treatment program nearly 67% of patients were in WHO clinical stage III and IV. More than half (59.2%) of patients were enrolled in the differed treatment program. In differed program, 178 (60%) of cases were initiated ART treatment with both WHO clinical staging and CD4 count. Majority of patients were on first line treatment regimen in both cases (Table 1).

### Survival status and treatment outcome

Five hundred patients were followed for different periods of time with a total of 1632.6 person-year of observation. During the follow up period, 48 patients died. Hence, the overall incidence density rate (IDR) of death in the cohort was 0.03 per person-year which is equal to 3 people per 100 peoples within 1 year of observation. It is significantly different for the two comparison groups. The incidence density rate was 0.038 per person year of observation in differed treatment groups whereas the incidence density rate was 0.024 per person year in universal treatment program with a p value of 0.001. The relative risk of death among differed cases was 1.58 times higher than the UTT cases.

**Fig. 1:**
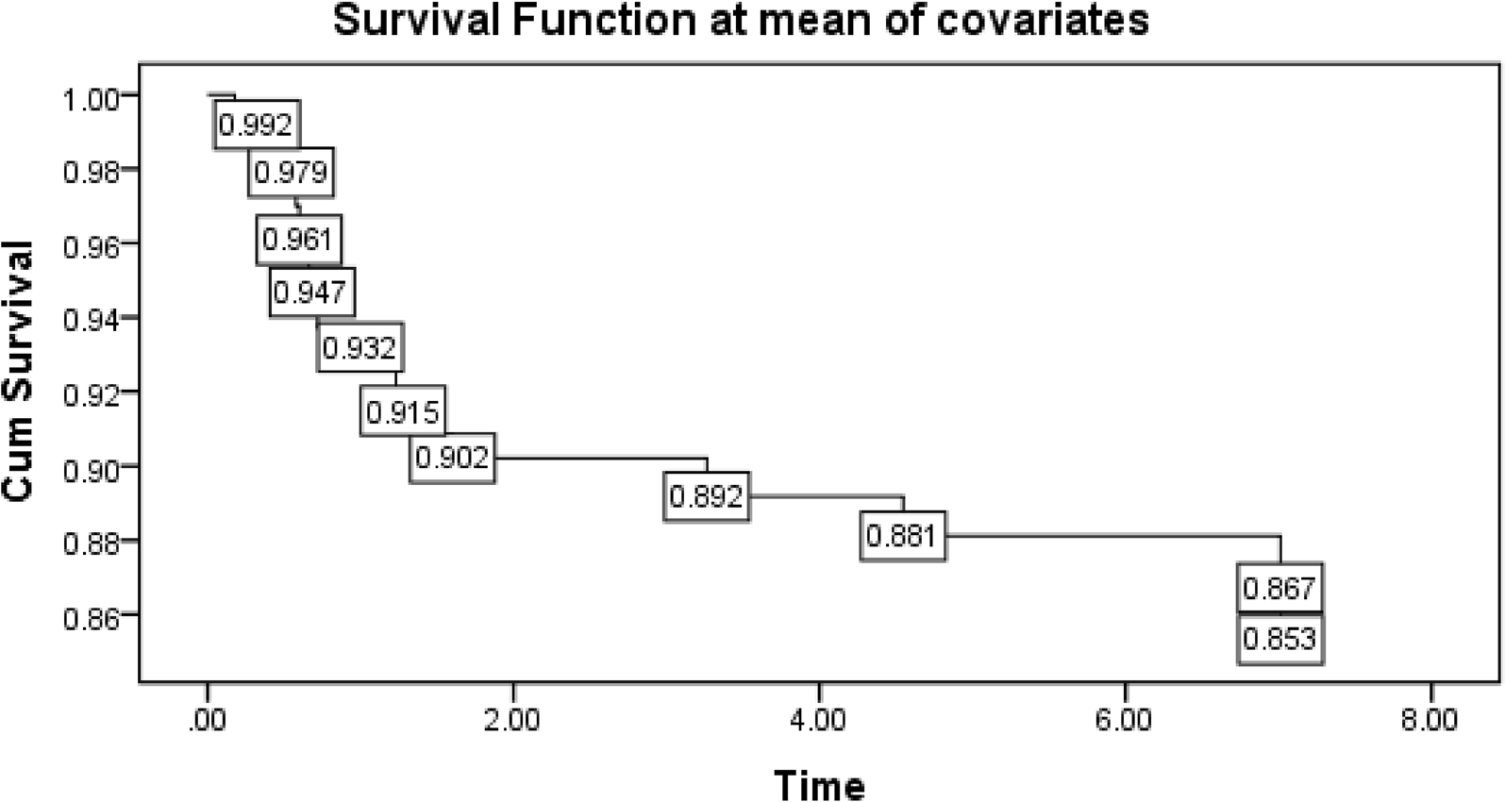
Cumulative survival estimate among HIV infected patients in cART program, Gurage zone, 2019

**Fig. 2:**
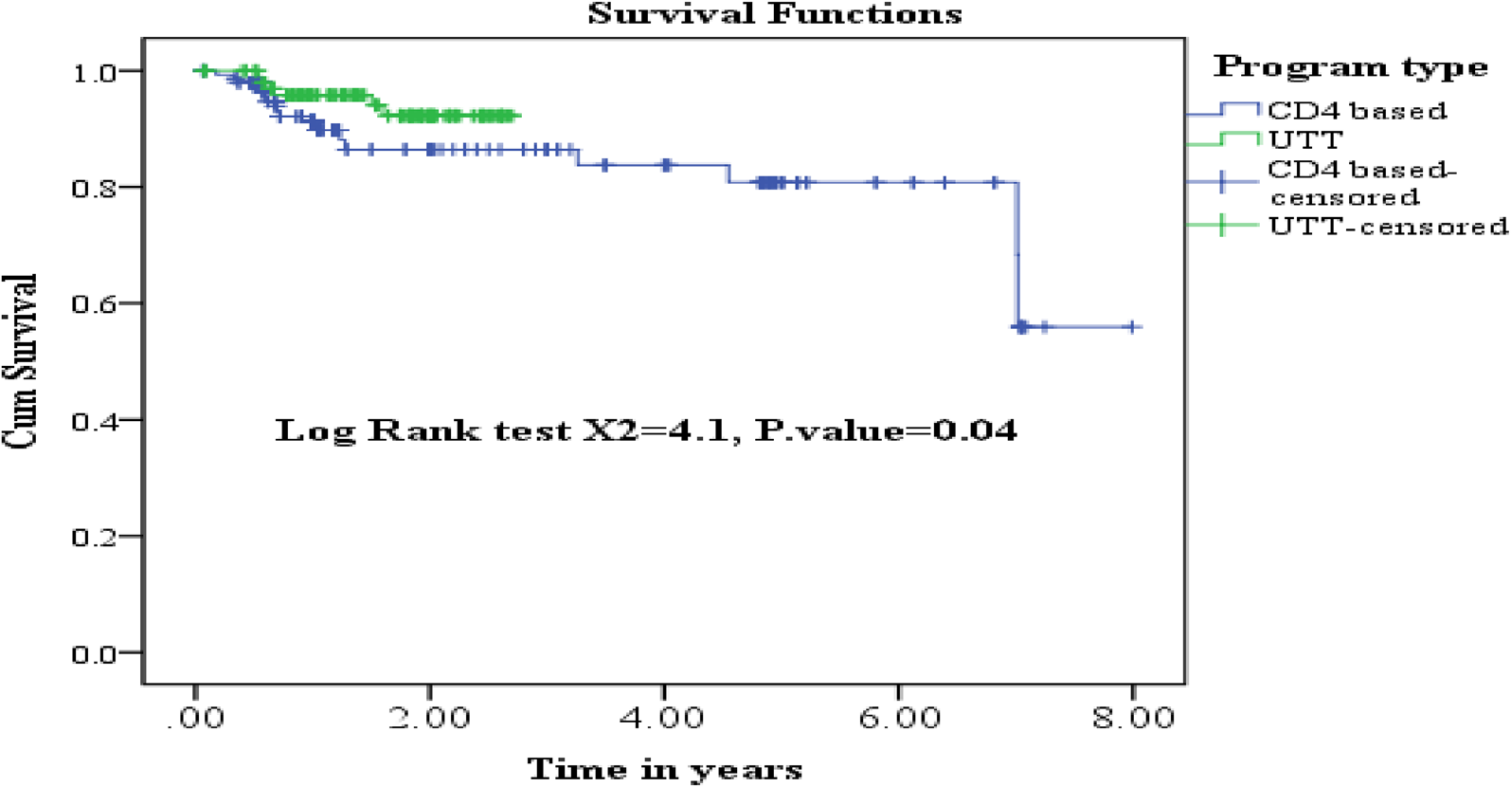
Kaplan–Meier survival estimate among HIV infected patients in cART program, Gurage zone, 2019

The cumulative probability of survival at the end of 1st, 2nd, 3rd, and 4th years of enrolment to treatment was 98%, 90.2%, 89.2% and 88% respectively with significant difference between the two groups. The log rank test and Kaplan–Meier survival curve indicated that a survival difference between the two groups is significant. However, the median survival time was undetermined. Because the largest observed analysis time was censored; the survivor function does not go to zero (Fig. 1). Patients enrolled in the UTT program survive longer than clients enrolled in the differed treatment program (log rank X^2^ test = 4.1, p value = 0.04) (Fig. 2).

During the follow up period 48 (9.6%) patients died, 44 (8.8%) dropped out from treatment program, 90 (18%) transferred out, and the remaining 318 (63.6%) patients are on treatment follow up. Most deaths were recorded within the first few years of treatment initiation (Table 2).

**Table 2:** Treatment outcome of HIV infected patients in the cART program, Gurage zone, 2019

Treatment outcome	UTT N (%)	CD4 based N (%)	Total N (%)
Alive	162 (79.4)	156 (52.7)	318 (63.6)
Dropped out	10 (4.9)	34 (11.5)	44 (8.80)
Transferred	20 (9.8)	70 (23.65)	90 (18.0)
Died	12 (5.9)	36 (12.15)	48 (9.60)
Total	204 (100)	296 (100)	500 (100)

### Factors associated with mortality

#### Bivariate and multiple cox regression

In bivariate cox regression, age, sex, educational status, base line weight, base line CD4 count, program of enrolment, development of new OIS and treatment failure were associated with mortality. By using variables which have p value less than 0.25 in the bivariate analysis multiple cox regression was fitted with forward stepwise method. After controlling the effect of other variables age, residence, base line CD4 count, program of enrolment, development of new OIS and treatment failure significant predictors of survival time or mortality of HIV patients who are on ART treatment (Table 3).

**Table 3:** Binary and multiple cox regression analysis of factors associated with death, Gurage zone, 2019

Predictors	Outcome		CHR (95% CI)	p value	AHR (95% CI)	p value
	Died	Censored				
Age	48	452	1.05 (1.01–1.08)	0.002	1.05 (1.01–1.08)	0.008
Sex						
Male	20	144	1			
Female	28	308	1.54 (1.13–2.08)	0.006		
Residence						
Rural	36	184	2.7 (1.73–4.2)	0.000	2.42 (1.5–3.8)	0.000
Urban	12	268	1		1	
Marital status						
Single	4	74	1			
Married	22	242	0.78 (0.47–1.08)	0.3	–	
Divorced	22	136	1.01 (0.92–1.21)	0.26	–	
Educational status						
Illiterate	26	168	1.35 (0.99,1.83)	0.05	–	
Literate	22	294	1			
Weight	48	452	0.95 (0.94–1.06)	0.9	–	
WHO stage						
Stage I	4	114	1			
Stage II	4	124	1.04 (0.13–5.3)	0.8	–	
Stage III	38	192	1.05 (0.12–4.1)	0.7	–	
Stage IV	2	22	1.1 (0.2–6.1)	0.9	–	
Program						
UTT	12	192	1		1	
Differed	36	260	3.45 (1.7–7.2)	0.001	4.13 (1.86–9.17)	0.000
New OIS						
Yes	12	28	2.87 (2.03–4.07)	0.000	3.66 (2.4–5.6)	0.000
No	36	424	1		1	
Treatment failure						
Yes	18	12	11.34 (5.8–22)		3.8 (1.8–8.4)	0.000
No	30	440	1		1	
Treatment switch						
Yes	2	6	1.2 (0.6–2.5)	0.6	–	
No	46	446	1			
Base line CD4 count	48	452	0.99 (0.99–1.1)	0.06	0.996 (0.993–0.999)	0.017

After controlling the effect of other variables, patients living in rural setups were 2.42 (95% CI [1.5–3.8], p value < 0.001) times more likely to die than urban resident patients. The risk of death was 4.13 (95% CI [1.86–9.17], p value < 0.001) times higher for patients who were enrolled in the differed treatment (CD4 based) program than patients enrolled in the universal test and treat program. Likewise, patients who developed treatment failure were 3.8 (95% CI [1.8–8.4], p value < 0.001) times more likely to die than their counter parts. Similarly, the risk of death in patients who developed new OIS was 3.66 (95% CI [2.4–5.6], p value < 0.001) times higher than those who did not develop new OIS. In addition, the increment of base line CD4 count by one unit reduces the probability of death by 0.4%. On the other hand the likely hood of mortality was increased by 5% as age increased by a year (Table 3).

## Discussion

This study assessed the effects of UTT program in comparison with the differed program on survival status and treatment outcomes of HIV infected patients initiated ART treatment in health facilities of Gurage zone. The incidence of death was significantly higher in the differed (CD4 based) program than the UTT program. It may be due to the fact that patients during the differed program commonly present with late WHO clinical stages or after developing serious opportunistic infection [2–6]. Similarly, the immune response depends on CD4 level, so that; patients in the differed program may not have good response to treatment [5].

Patients in the UTT survived for longer period of time than patients enrolled in the differed treatment program. As the universal test and treat program makes patients to get medical support in the early stages of infection the response to treatment will be obviously better [7–9]. Meanwhile, early treatment and prophylaxis prevents the development of fatal opportunistic infections. So that the survival of patients in the test and treat program is longer [2, 3]. Also previous studies reported that early presentation and medical care increases the survival of patients [14–18]. It has to be noted that patients who were enrolled under differed program were not followed until their CD4 count or WHO stage make them eligible to be enrolled for treatment. However patients on UTT program were enrolled for treatment soon after diagnosis, hence these time lapses between diagnosis and enrolment for treatment would have an impact on survival time differences.

The probability of survival at the end of 2 years of follow up is higher than the findings of previous studies conducted elsewhere [15, 17–19]. This is due to the effect of the universal test and treat program included in our study, which increases the survival of patients [16, 19]. On the other hand, the cumulative incidence of mortality was significantly higher among patients enrolled in differed treatment programs compared to patients enrolled in UTT program. This can be explained by increased risk of opportunistic infections, treatment failure and drug side effects which are more common in the differed treatment arm [15–18].

During the follow up period 48 (9.6%) patients died. Lower rate of death was observed in the UTT cohort. The proportion of death in our case is lower than many other studies [17, 20, 28]. There are also other recent researches that have reported a lower AIDS related death rate in Ethiopia [29–32]. This could be partly attributed to the effects of UTT program implementation in Ethiopia.

Change in treatment regimen, patient condition during admission, program organization, residence and multitude of other factors significantly contribute for the difference in survival rate. With all this benefits early initiation of treatment with UTT program reduced mortality [17, 28]. Generally, in Ethiopia it was noted from WHO reports and previous studies that the success rate was higher in both of HIV treatment programs but more positive outcomes are found on UTT program [1, 2, 28].

In line with finding of this study, many other model based researches have reported that UTT program will reduce mortality [16, 17, 31]. We hazard of death in CD4 based enrolled individuals was 4 times higher than those who enrolled in UTT program. This is in line with other studies [25–27, 33]. This could be due to the fact that UTT program clients are enrolled for treatment soon after diagnosis when many of them are at higher CD4 count and better overall health conditions [17, 26]. This could result in low prevalence of co-infection, low probability of drug interaction and side effects and overall better compliance. Owing to the aforementioned positive attributes of UTT program the hazard of mortality has reduced.

Patients living in rural setups were two and half times more likely to die than urban resident patients. This may be due to better drug adherence, accessibility of service, and knowledge difference. Likewise, patients who have developed treatment failure were four times more likely to die than their counter parts. In many researches it was reported that treatment failure is a strong marker of mortality while up on treatment [3, 15, 17]. This may be due to increased viral load and development of secondary infections [22, 33].

Similarly, the risk of death in patients who developed new OIs was 3.66 (95% CI [2.4–5.6], p value < 0.001) times higher than those who did not develop new OIs. Also the increment of base line CD4 count by one unit reduces the probability of death by 0.4%. On the other hand the likely hood of mortality was increased by 5% as age increased by a year.

## Strength and limitation of the study

This research evaluated the impact of UTT in clinical setups, which may be the first to do so in the country. Therefore, it may help to know the case in the real scenario. Since the outcome is death; it is easy to establish temporal relationship with predictor variables that are documented at time of admission. On the other hand, incompleteness of information and reliability of the recorded data remains a major concern, since the data is obtained from record review. Also, facility related factors were not assessed in the study.

## Conclusion and recommendations

In this study the overall incidence density rate (IDR) of death in the cohort is lower than other studies. Similarly, IDR is lower in clients enrolled by UTT program. The cumulative probability of survival and overall mean survival time is higher in the UTT program and the overall value is comparable with other researches. Treatment outcomes measured in terms of favorable outcome (alive on treatment), death, and default rate were comparable to other reports as well. The main predictors of mortality were age, residence, base line CD4 count, program of enrolment, development of new OIs and treatment failure. Therefore, intervention to further reduce deaths has to focus on facilitating the UTT program to initiate treatment as early as possible and prevention of new OIs and treatment failure is needed. The finding of this research may provide necessary information in areas of improvement; however further research is needed to give policy level recommendations.

## Data Availability

Data is available and can be found upon request of the corresponding author.

## Abbreviations

ART: Antiretroviral therapy
AOR: Adjusted odds ratio
cART: Combined antiretroviral therapy
CI: Confidence interval
HIV: Human immunodeficiency virus
IDR: Incidence density rate
IRIS: Immune reconstitution inflammatory syndrome
OIs: Opportunistic infections
PLWHA: People living with HIV/AIDS
UTT: Universal test and treat
WHO: World Health Organization
